# Anti-dermatophytic activity of cold atmospheric plasma against *Trichophyton rubrum* via affecting fungal growth, morphology, drug susceptibility and *HSP90* gene expression

**DOI:** 10.1038/s41598-022-13828-4

**Published:** 2022-06-08

**Authors:** Asal Safi-Samghabadi, Seyed-Mohammad Atyabi, Mehdi Razzaghi-Abyaneh

**Affiliations:** 1 Department of Biology, Science and Research Branch, Islamic Azad University, Tehran, Iran; 2 Department of Nanobiotechnology, Pasteur Institute of Iran, Tehran, 1316943551 Iran; 3 Department of Mycology, Pasteur Institute of Iran, Tehran, 1316943551 Iran

**Keywords:** Biological techniques, Microbiology, Molecular biology

## Abstract

*Trichophyton rubrum*, a major human pathogenic dermatophyte, is responsible for the most recurrent dermatophytoses as globally important superficial fungal infections. Typical chemotherapy is used to handle such infections; however, emerging drug resistance and side effects necessitate the new remedial method development. Cold atmospheric plasma (CAP) is an emerging technology, consisted of neutral and charged particles and photons newly developed as a potent and safe antimicrobial technique to combat drug-resistant microbial pathogens. In the present study, the vast effects of CAP irradiation containing oxygen (2%) and helium (98%) on *T. rubrum* growth and pathogenicity were explored. After exposure of *T. rubrum* to CAP jet for 90, 120, 150, 180, and 210 s in 96-well microtiter plates, cell morphology and viability, ergosterol content of fungal hyphae, *HSP90* gene expression, and the pattern of drug susceptibility were studied by using electron microscopy, RT-qPCR, spectrophotometry, disk diffusion and CLSI microbroth dilution methods. CAP irradiation significantly inhibited the fungal growth by 25.83 to 89.10%, reduced fungal cell viability by 11.68 to 87.71%, disrupted cellular membranous organelles and structures of the fungal hyphae, and suppressed efficiently the expression of *HSP90* gene by 2 folds in 210 s exposure. Taken together, our results demonstrated that CAP is an efficient tool with potential *in-vivo* therapeutic applications against chronic dermatophytosis caused by *T. rubrum* due to its effectiveness, harmless, and ease of access.

## Introduction

Dermatophytes as specialized keratinophilic fungal pathogens are the leading cause of skin, nail, and hair infections globally, under the similar name of “dermatophytosis (Tinea, Ringworm) which affect about 25% of the world population^1^. Superficial and subcutaneous involvement of keratinized tissues and mucosal membranes are the prominent features of the disease in human and animals. According to the new classification, dermatophytes are classified into seven closely-related genera including *Trichophyton*, *Epidermophyton*, *Nannizzia*, *Paraphyton*, *Lophophyton*, *Microsporum*, and *Arthroderma*^2^.

*Trichophyton rubrum* is one of the major human pathogenic dermatophytes, accounting for approximately 69.5% of chronic dermatophytoses in humans. This pathogen particularly affects the immunocompromised patients, including those who are undergone long-term steroid therapy, bone marrow or solid organ transplantation, and patients with diabetes mellitus. *Trichophyton rubrum* relies on various virulence factors to initiate the infection. Most of the virulence factors are under the restrict control of regulatory elements such as heat shock proteins (Hsps). *HSP90* is a ubiquitously expressed chaperone involved in the proper folding of various protein kinases, nuclear receptors, and transcription factors. By regulating its target proteins (viz. protein kinases, nuclear receptors, and transcription factors), *HSP90* plays crucial roles in the maintenance of cell homeostasis. *HSP90* has an inevitable role in pathogenesis and life cycle of *T. rubrum*^3–6^. Considering the crucial role of *HSP90*, any modification in expression pattern of the protein has a vast effect on viability, morphology, and pathogenicity of the organism.

Treatment of dermatophytosis has gained new challenges due to the emerging dermatophytes with high resistance to current antifungal therapies in recent years^7,8^. Allylamines (terbinafine), azoles (ketoconazole and fluconazole) and griseofulvin are the choice drugs for treatment of dermatophytosis. Epidemiological changes, treatment failure due to drug resistance, long-term treatments, and side effects of antifungal drugs, have highlighted the significance of developing novel therapeutic methods with fewer side effects^9,10^.

Cold atmospheric plasma (CAP) is an emerging technology that is employed as a powerful tool with various applications in modern medicine^11^. CAP is a partially ionized gas that could be produced at atmospheric pressure. Since the particles are not in thermal equilibrium in the CAP, they can operate close to the room temperature^12^. CAP includes a highly reactive combination of ions, electrons, reactive molecules (reactive oxygen species, ROS; reactive nitrogen species RNS), excited species, electric fields, and UV radiation. It has been reported to exhibit antimicrobial, antifungal, anti-biofilm, wound healing, and anti-cancer effects^11–15^. This method could be considered as a safe treatment option, since no side effects have yet been reported on healthy cells.

The present study aimed to investigate the mode of action of CAP irradiation toward the growth and pathogenicity of *T. rubrum*, with special focus on *Hsp90* gene expression (a key fungal pathogenicity biomarker), fungal cell morphology and viability at electron microscopy, ergosterol content of hyphae cell membrane and the pattern of susceptibility to current antifungal drugs.

## Results

### Inhibitory activity of CAP on *T. rubrum* growth

**Figure 1:**
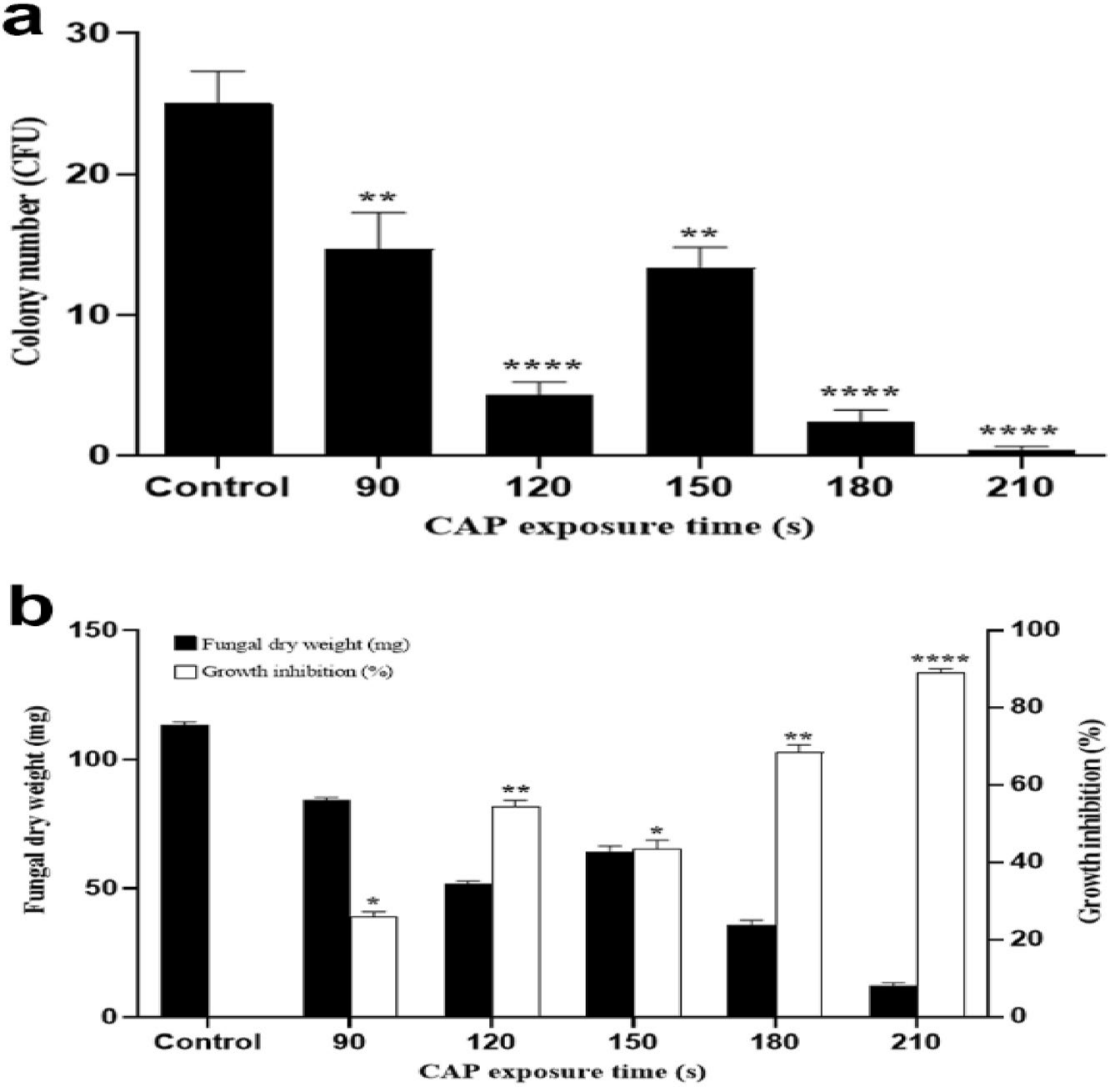
Colony number and dry weight of *T. rubrum* after CAP treatment for 90, 120, 150, 180, and 210 s in comparison to control group. The fungus was treated with CAP in three separate experiments and the results are represented in the graphs. (**a**) Number of *T. rubrum* colonies (**b**) Inhibition percentage of dry weight and growth. The error bar indicates the standard deviation. Asterisks show statistically significant difference with a control determined by ANOVA (* *P* < 0.05, ** *P* < 0.01, *** *P* < 0.001, **** *P* < 0.0001).

To investigate the effect of CAP on fungal growth, the colony counting approach was performed (Table 1). Treated samples in all CAP exposures showed a significant reduction of colony count. The average number of colonies in the untreated control sample was 25.0 which reduced to 14.66 at 90 s, 4.33 at 120 s. 13.33 at 150 s, 2.33 at 180 s and 0.33 at 210 s CAP exposure. Therefore, increased plasma flow time, significantly decrease the number of fungal colonies (Fig. 1a). A significant reduction in colony growth occurred during 180 s irradiation with an average of 2.33. Ultimately, after 210 s of plasma exposure, the number of colonies fell into 0.33. As shown in Fig. 1a, a significant difference between the treated and untreated samples was evident (*P* < 0.05). The Fig. 1b shows that the CAP exposure over 210 s significantly inhibited the fungal growth in a dose-dependent manner.

**Table 1:** Effect of CAP on the fungal growth and antifungal activity of terbinafine and ketoconazole against *T. rubrum.*

Plasma treatment (Time; s)	Colony number (CFU) (Mean ± SD)	Micro dilution assay (MIC; μg/mL)		Disk diffusion assay (zone of inhibition; mm)	
		Terbinafine	Ketoconazole	Terbinafine	Ketoconazole
Control	25.0 ± 4.0	1.0	2.0	7.60	7.06
90	14.66 ± 4.50*	0.5	1.0	8.24*	9.72*
120	4.33 ± 1.52*	0.125	0.25	10.52*	10.08*
150	13.33 ± 2.5*	0.25	1.0	10.40*	9.74*
180	2.33 ± 0.52*	0.0625	0.25	10.58*	10.12*
210	0.33 ± 0.07*	0.0625	0.0625	10.80*	11.20*

*Statistically significant difference with a control (ANOVA, *P* < 0.05).

As shown in Fig. 1b, the average fungal dry weight in untreated samples was 113.3 mg. With the plasma flow of 90 s and 120 s, the dry weight of the samples decreased to an average of 84.0 (25.83% inhibition) and 51.5 mg (54.47% inhibition), respectively. With 150 s of treatment, the dry weight was determined to be 63.9 mg (43.54% inhibition), which was slightly increased compared to 120 s of exposure. Nonetheless, this average of dry weight was meaningfully lower than the control samples. In the 180 s time of exposure, the dry weight reached the average of 35.7 mg (68.42% inhibition) and continued in a decreasing mode to 12.4 mg (89.1% inhibition) in the CAP flow of 210 s. The differences were statistically significant between the dry weights of the control and each of the treated samples (*P* < 0.05).

### In vitro cell cytotoxicity of CAP

**Figure 2:**
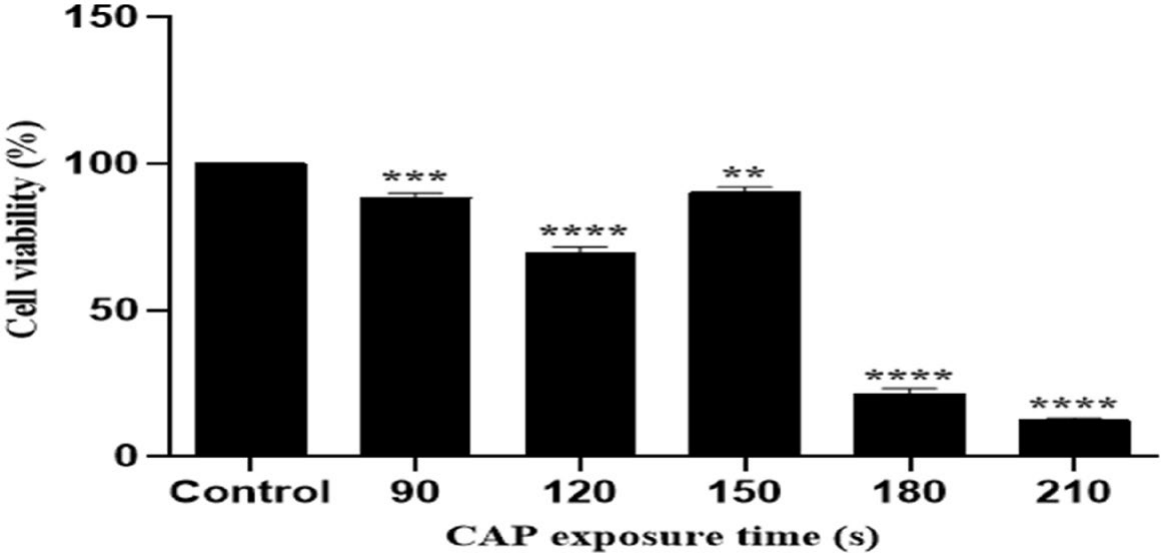
The MTT cell viability assay of CAP treated *T. rubrum* cells exposed to CAP for 90, 120, 150, 180, and 210 s. The MTT assay was done 24 h after CAP treatment for different CAP exposure times (X-axis) and the percentage of cell viability (Y-axis), determined by colorimetry (density of purple color of formazan) and represented as vertical bars. The error bars show the standard deviation. Asterisks show statistically significant difference with the control group (ANOVA, ** *P* < 0.01, *** *P* < 0.001, **** *P* < 0.0001).

Figure 2 shows fungal cell viability of *T. rubrum* exposed to CAP by the MTT test. The percentage of viable cells in the samples with 90 s and 120 s of CAP treatment were 88.3% and 69.2%, respectively; the cell viability was significantly decreased in comparison with the control group. In contrast, 150 s treated samples showed the viability of 90.2%. The fungal survival rate reached to 21.6 and 12.3% in 180 s and 210 s treated samples, respectively.

### Effect of CAP on ultrastructure of *T. rubrum*

**Figure 3:**
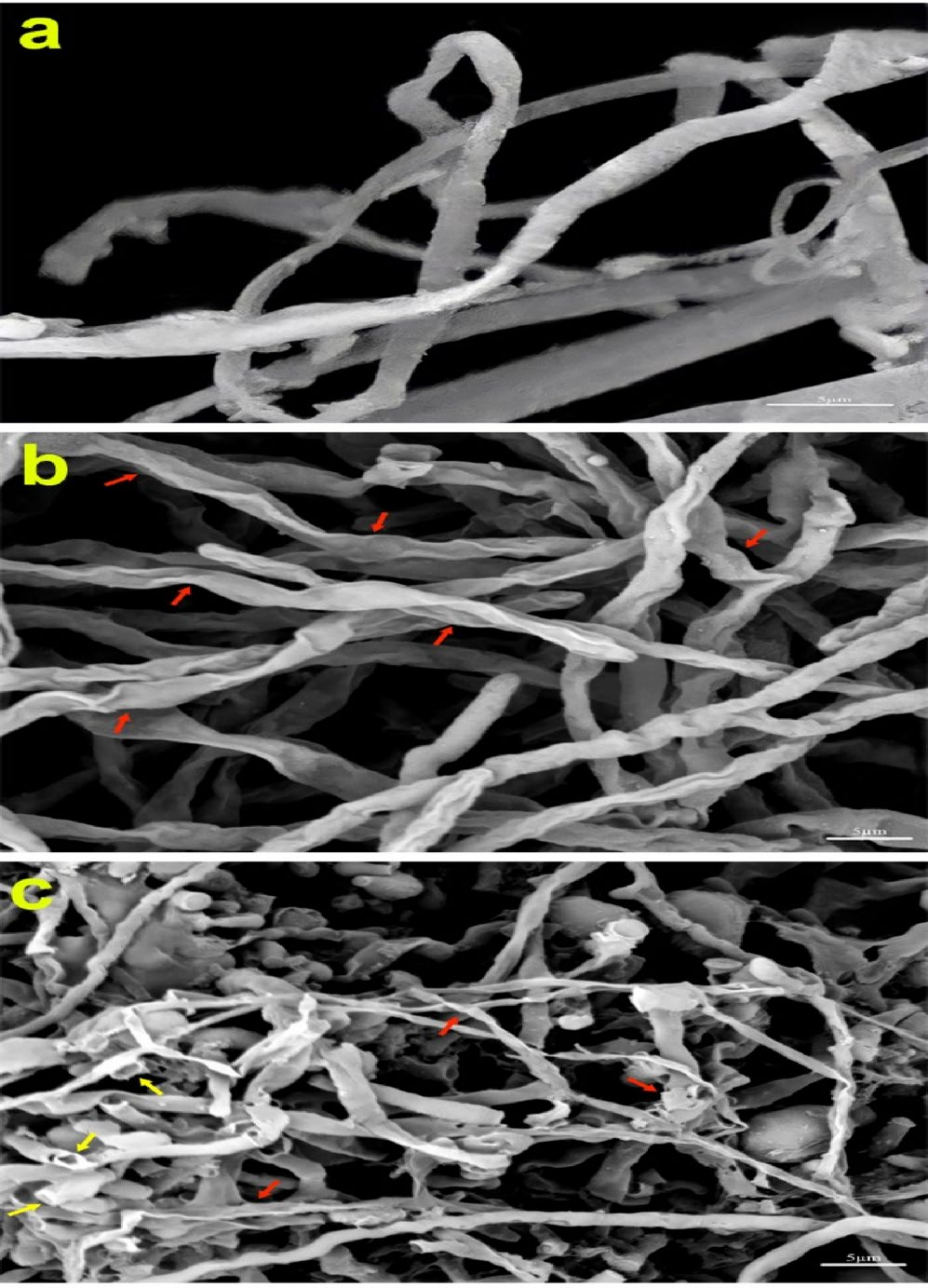
Scanning electron microscopy of *T. rubrum* morphologies. Images represent the cells in non-treated (**a**) and CAP-treated samples (**b** and **c**). The untreated sample (control) shows normal tubular and uniform hypha. The cells were treated by CAP irradiation for 210 s and cultured for 24 h (A) and 96 h (B). There is minor deformation in the shape of the cells and break down in cell wall or wrinkle after 24 h of cultivation (red arrows) (**b**), while significant alterations are evident after 96 h incubation (**c**); these are the fragmentation of cells into flat shapes (red arrows), the leakage of intracellular material after perforation (yellow arrows).

Figure 3 shows morphological changes of fungal hyphae exposed to CAP. The scanning electron microscopy (SEM) images of the control group showed completely normal cells characterized by tubular filamentous hyphae, smooth rigid cell walls, and intact cell integrity. The deformed cells were observed 24 h after CAP treatment for a maximum of 210 s. The deformation included the cell wall breakdown, which was appeared as scattered hypha. After 96 h of incubation, the changed mycelial structures were more discernable; folding of fungal hyphae, increased cell permeability (defined by leakage of intracellular material), and formation of spherical bulges are among the most important observed changes. Moreover, filamentous hypha transformed to flat shapes (Fig. 3).

**Figure 4:**
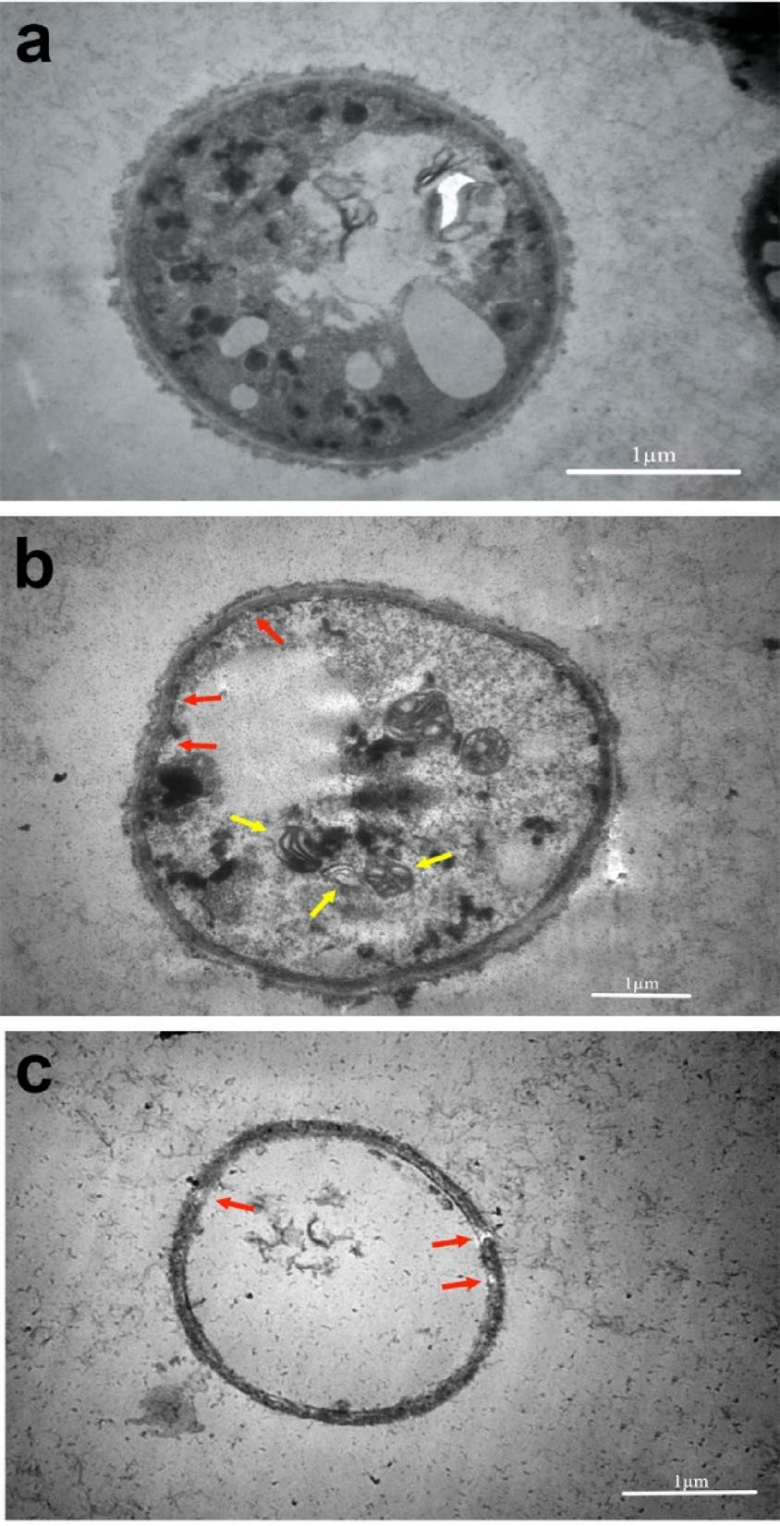
Transmission electron microscopy of *T. rubrum* cells in non-treated (**a**) and CAP-treated (**B**-**C**) samples. The images were taken from control samples (**a**), and 210 s CAP treated samples after 24 h (**b**) and 96 h (**c**) incubations. A typical cell is represented by control sample. There are noticeable changes as cytoplasmic degradations, deformation of the cells and intracellular organs in B especially mitochondria (yellow arrows) and deformation of cell membrane (red arrows) while the severity of cell damages are obvious in C as breaking down of intracellular organelles into larger vesicles, deformation of cells and the membrane became more detached from the fungal cell wall.

Transmission electron microscopy (TEM), on the other hand, unveiled internal changes upon CAP treatment and provided more details on the cell organelles, cell walls, and cell permeability. After treatment for 24 h, the internal changes were observable, including the morphological transformation of the cells, changes in organelles, and nucleus destruction. Damage severity continued to increase until 96 h. After 96 h, the cells were completely deformed, cell walls became thinner, and cell organelles became broken down. These cell distractions were more apparent in cells of 210 s exposure (Fig. 4).

### Effect of CAP on *T. rubrum* cell membrane ergosterol synthesis

**Figure 5:**
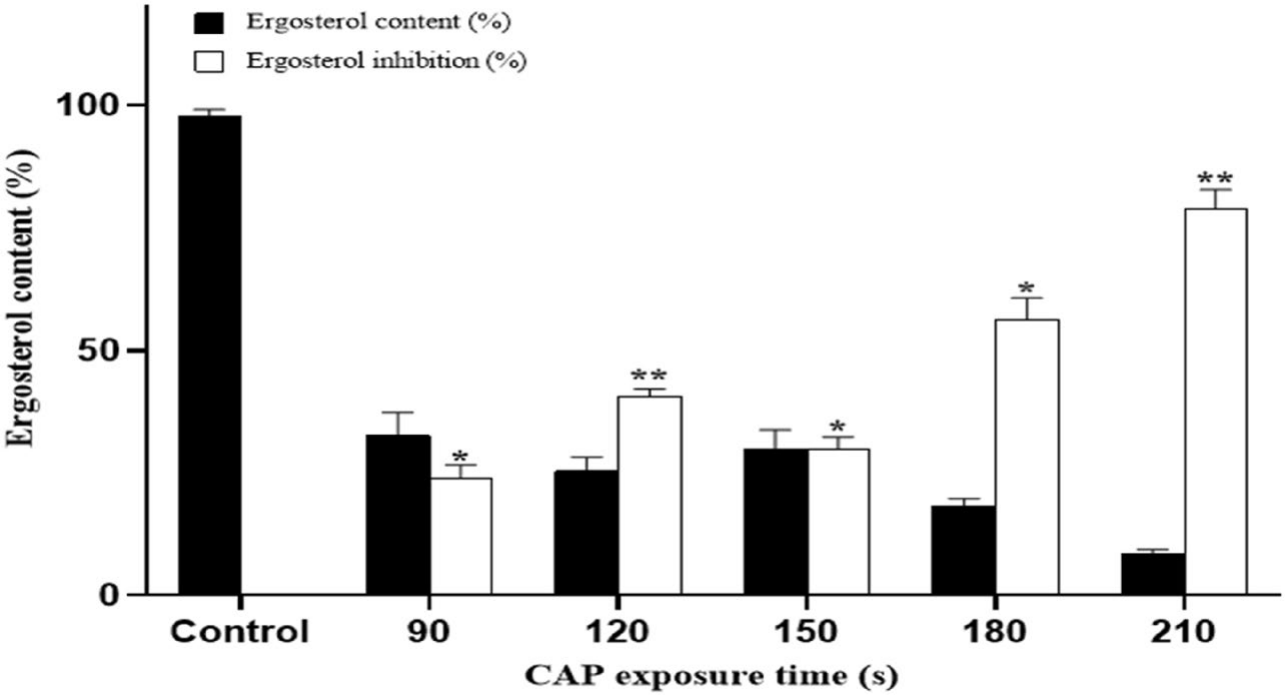
Ergosterol content of *T. rubrum* after CAP treatment for 90, 120, 150, 180, and 210 s in comparison to control group. The fungus was treated with CAP in three separate experiments and the results are represented in the graphs. The X-axis in figure shows the time of CAP exposure and the Y-axis is the analyzed Ergosterol content percentage. The error bar indicates the standard deviation. Asterisks show statistically significant difference with a control determined by ANOVA (*P < 0.05, **P < 0.01, ***P < 0.001, ****P < 0.0001).

Spectrophotometry was exploited to measure the amount of ergosterol in each dried mycelial extract (Fig. 5). There was a statistically significant difference between various CAP treatment times (*P* < 0.05) in the terms of ergosterol biosynthesis. The percentage of ergosterol in the control sample was 42.66%. This level was decreased to 32.66% and 25.33% in plasma-treated samples of 90 and 120 s, respectively. This value was slightly increased (30%) for the samples with 150 s of treatment and then continued to decrease for samples with 180 and 210 s of treatment and reached 18.33% and 8.66%, respectively. The ergosterol level decreased in all treatment durations compared to the control samples. As is evident in Fig. 5, increased plasma exposure, decreased the ergosterol biosynthesis by 24–80%. Although a fluctuating profile was observed during plasma irradiation in every 30 s, the overall decrease in ergosterol levels was clear after 210 s of treatment.

### Effect of CAP on *HSP90* gene expression in *T. rubrum*

**Figure 6:**
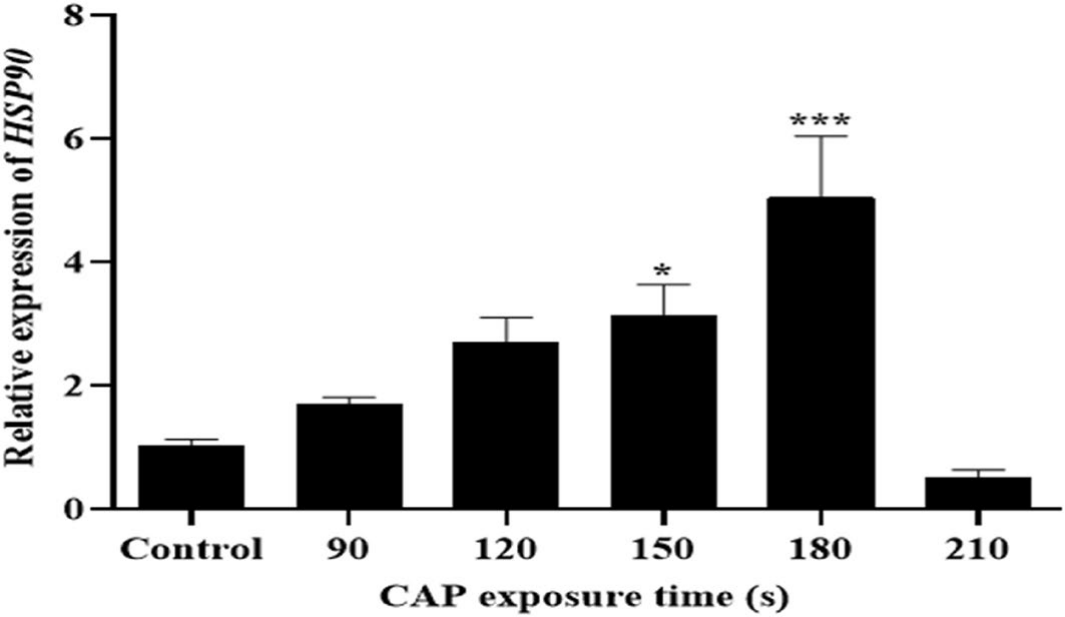
The expression levels of heat shock protein 90. RT-qPCR analysis of RNA levels in *HSP90* gene in *T. rubrum* expose to CAP in different times of 90, 120, 150, 180, 210 s and control sample. Error bars represent standard deviation. Asterisks show statistically significant difference with a control (ANOVA, *P < 0.05, ***P < 0.001).

As a characteristic of stress condition, the expression pattern of the *HSP90* gene was evaluated in control and test groups by a two-step RT-qPCR. The sequence of *HSP90* gene of *T. rubrum* was deposited in the GenBank under the accession number OM994407. The graph in Fig. 6 depicts the differences in expression patterns of the *HSP90* gene in various groups; the highest expression occurred in samples with 180 s of CAP treatment (fivefold), while this value was significantly dropped in the samples with 210 s of CAP treatment (almost half the amount of control).

### Effect of CAP on *T. rubrum* antifungal drug susceptibility

As shown in Table 1, disc diffusion and MIC approaches evaluated the effect of CAP treatment on the susceptibility of *T. rubrum* to Terbinafine and ketoconazole. While the inhibitory zones of the mentioned drugs were respectively 0.80 and 0.53 in diameter for the control group, the CAP treated samples showed increased susceptibility defined by wider inhibitory zones with the highest diameter observed in 180 s and 210 s treated samples. The samples with 180 s and 210 s of CAP treatment were both more sensitive to Terbinafine. With ketoconazole, the maximum effect was observed in the group with 210 s of treatment. The lowest MIC of Terbinafine was 0.0625 μg/mL, which was observed in groups with 180 s and 210 s of CAP treatment. This index was 0.0625 μg / mL for ketoconazole, which was achieved in the 210 s CAP treated group (Table 1).

## Discussion

Drug resistance remains to be a tough challenge ahead of ongoing treatments against microbial pathogens and demands for novel therapeutic approaches to enhance the effectiveness of existing medications. The present study demonstrates the effect of CAP treatment on the growth and pathogenesis of *T. rubrum* at cellular and molecular levels. Ebrahimi-Shaghaghi et al.^11^ showed that CAP efficiently inhibited *Candida albicans* growth and *HSP90* gene expression in the fungus. In the present study, the growth of *T. rubrum* was inhibited in CAP treated samples in relation to decrease in fungal cell membrane ergosterol and cell viability. The fungal growth was decreased meaningfully in all CAP treatments with an slightly increase in the exposure time of 150 s. In 150 s after CAP treatment, the number of colonies were slightly increased (Fig. 1) but it still was significantly lower than that of the control samples. This may be a consequence of fungal response against cell death under the stress of CAP treatment. With increasing the treatment time to 180 s and 210 s, this ability was gradually lost due to the significant fungal cell death. This is in accordance with the results of Ebrahimi-Shaghaghi et al.^11^ who reported a similar behavior for CAP-treated *Candida albicans* cells. Ergosterol, as the unique lipid of the fungal membrane, has a critical role in the structure and fluidity of fungal membranes; additionally, it is an immunologically active molecule^17–19^*.* So, reduction of its biosynthesis would decrease the mycelial weight as observed in our study. These initial impairments and vast organelle destruction might explain the subsequent reduction of cell viability. Bekeschus et al.^20^ have investigated the effect of CAP treatment on the *HSP90* in cancer cells. They have reported that the pro-apoptotic effects of CAP treatment could be attributed to its effect on *HSP90* expression.

The CAP-induced impairments of the membrane structures of *T. rubrum* were evident in SEM and TEM images in the present study. Since the majority of morphological disorders are due to membrane peroxidation as a general rule, the broader damages to the organelles of the fungus could be explained by the oxidative reactions of species of CAP to all membrane-enveloped structures. Our results is consistent with the results of He et al.^21^, which examined the effect of CAP on cell membranes of glioblastoma multiforme. They showed that CAP-induced endocytosis might be the reason for the observed vesicular budges.

Membrane damage is known to induce ROS production through coenzyme Q redox cycling^22^. This excessive ROS may foster the oxidative effect of CAP irradiation. The endogenous production of reactive species was reported in the study of glioblastoma, an extremely aggressive brain tumor, through inhibition of glutathione/glutathione peroxidase^23^. The authors also mentioned the usefulness of CAP combination with chemotherapy. It has also been shown that CAP treatment could lead to changes in the activity of membrane proteins^24^. These changes could be deemed as the other explanation for the morphological changes through CAP irradiation. However, the damage is seemingly not limited to the membrane-embedded proteins.

The increasing effect of anti-fungal drugs observed in this study might be attributed to the *HSP90* inactivation, since a strong correlation has been reported in CAP treatment and increasing drug efficacy^25^. Increasing time of CAP exposure showed to possessed a significant effect on the expression level of the *HSP90* gene^25^. The expression level increased until the 180 s of treatment, at higher treatment durations the cell began to die. The signs of cell death appeared which is consistent with harsher damages induced by the time of irradiation. The time of exposure to the CAP irradiation has implications on cell death^26^. CAP exposure time or excessive higher intensity may broaden the consequence of irradiation. The highest effects were seen in longer durations (these are 180 s, 210 s). It has been shown that short treatment times with CAP (30–120 s) failed to cause DNA damage in maize grains. On the other hand, longer irradiation may cause a higher extent of damage to DNA and proteins^27^. Therefore, it does not strike as a surprise that we detected the more harmful effects in longer durations of CAP treatment.

Terbinafine belongs to allylamines and ketoconazole, a broad-spectrum imidazole antifungal drug, are shown to interfere with ergosterol biosynthesis pathway^28,29^. The CAP exposure, on its own, can influence the ergosterol biosynthesis. Likewise, our results showed that drug susceptibility of *T. rubrum* to both terbinafine and ketoconazole was increased in the fungus exposed to CAP in a time-dependent manner. In a recent study by Ebrahimi-Shaghaghi et al.^30^, CAP was successfully used for treatment of vulvovaginal candidiasis in a murine model. So, a combination of CAP treatment with both drugs may have a synergistic effect would result in a wider range of fungal cell membrane destruction and decrease the effective dose of these antifungal drugs in vivo.

Due to the nature of *T. rubrum* infections which is comprised of a complex mixture of species^31,32^, the generalizability of this study is somehow limited, nevertheless due to the extreme genotypic similarity^33^ the observed results could be expanded to other species. Several questions remained unanswered about the reactive species and the precise influence of CAP irradiation on various critical virulence factors, gene regulatory elements, and membrane determinants. Therefore, there is abundant space for future progress by focusing on delivering the reactive species to the right place, in the right concentration, and at a right time. For example, the heat shock proteins are sustained as critical components in the life cycle, adaptation, and pathogenesis of dermatophytes^34,35^, it would prompt future research on the effect of CAP on other members of this big family. To add, ergosterol biosynthesis in an extensively complicated pathway, for which at least 20 genes have been identified, each of which could be affected by CAP irradiation, hence are attractive subjects for more specific studies.

## Conclusion

The relentless trait of drug resistance has forced researchers to search for novel therapeutic approaches to manage the infectious complexities; the method that is efficient, cost-effective, and easy to access. Such an approach should fulfill the consistency by which the resistance development should be ruled out. Tangled sequential events were seen after CAP treatment, which are extensively under control of *HSP90*. CAP exerted its action on multiple components of *T. rubrum* hyphae including cell membrane and gene expression patterns through the production of reactive radicals and induction of oxidative stress; these all-together aid the infection therapy by direct killing the fungal pathogen on superficial lesions of infected patients or by increasing drug susceptibility indirectly. Although the cold atmospheric plasma is on its initial steps, the technology is growing in a fast pace, providing an open window for new research field and hope is sparking for the technique to adapt to specific therapeutic applications.

## Materials and methods

### Fungal strain and culture medium

The pathogenic strain of *Trichophyton rubrum* HR1 (IR 613; PFCC 51431) was obtained from the Pathogenic fungi Culture Collection of the Pasteur Institute of Iran (http://fa1.pasteur.ac.ir/VisitDetails.aspx?Id=1605). The fungus was cultivated on Sabouraud dextrose agar (Merck, Germany) enriched with chloramphenicol (0.005%) and cycloheximide (0.04%), and incubated at 28 ºC for two weeks.

### Preparation of fungal conidia suspension

The uniform conidia suspension of *T. rubrum* was prepared as described previously^14^. Briefly, adequate amount of sterile distilled water contained tween 80 (0.1%) was added to the fungal culture in slants to disperse the conidia throughout the culture. The conidia suspension was prepared by gentle rubbing of culture surface by a sterile glass rode and then passed through cheese cloths to remove hyphae fragments and remaining debris. The number of conidia in the clarified suspension was counted by a hemocytometer. The conidia suspension was kept at room temperature until use.

### The instrument conditions and CAP treatment

The experimental setup for CAP generation, included a helium gas cylinder, rotor flow meter, power controller, and plasma jet. A mixture of oxygen (2%) and helium (98%) was used. The peak-to-peak voltage applied to the electrode was set as 10 V, the sinusoidal wave frequency was set to 15 MHz, the output power was set to 10 W, and the flow rate of He gas was set to one L/min. A manometer (gauge) was used to regulate the output pressure of the gas. The temperature for Helium–Oxygen (He/O_2_) was 28 ºC. At each exposure time, the distance was two cm between the plasma jet nozzle and the sample surface. For each experiment, 50 µL of the suspended fungal conidia were divided into 6 columns of 96-well plates^36^. The treatments were carried out in triplicate through exposing fungal conidia to the cold atmospheric plasma jet for 0, 90, 120, 150, 180, and 210 s.

### Determining the fungal mycelia weight

One-hundred µL of a 2 × 10^6^ cells/mL conidia suspension was dropped into the selected wells of 96-well sterile microtiter plates. The plates were treated with CAP as described earlier, for 0, 90, 120, 150, 180, and 210 s. The treated samples were inoculated into the Sabouraud maltose broth (E-Merck, Germany) and incubated for 3 days at 150 rpm at 37 ºC. After incubation, the dry weight of fungal biomass was measured. Fungal mycelia was separated from the culture medium using Whatman filter paper No. 1. After 6-h incubation at 60 ºC, the weight of dried fungal biomass of treated and control samples was measured and compared^14,37^. The growth inhibition percentage based on dry weight was calculated as:

$$\left( {{\text{Control weight}} - {\text{Sample weight }}/{\text{ Control weight}}} \right) \, \times \, 100.$$

### Post-CAP-treatment assessment of fungal cell membrane ergosterol

One-hundred μL of the CAP treated and non-treated fungal conidia were inoculated in 30 mL Sabouraud maltose broth in 100 mL Erlenmeyer flasks and maintained into a shaker incubator for 72 h at 30 °C. Fungal mycelia was separated from the culture medium using Whatman filter paper No. 1, washed three times with distilled water and dried for 3 h at 60 ºC. The dried biomass for all treatments in equal weight was mixed with 25% alcoholic potassium hydroxide solution with a Vortex mixer and incubated in a water bath of 80 °C for 1 h. Total contents of the sterols were extracted by addition of 1 mL of distilled water and 3 mL of *n*-hexane to the samples and their vigorous vortex for 1 min. The *n*-hexane phase containing the sterols was separated and stored at − 20 °C for 24 h. Afterward, the samples were diluted 5 folds with absolute ethanol and scanned with a spectrophotometer (EZ 301, Perkin-Elmer, USA) at 200 nm and 300 nm. The following equation^38^ was invoked to calculate the amount of ergosterol:

$${\text{Ergosterol }}\left( \% \right) \, = \, \left[ {\left( {A_{281.5} / \, 290 \times F} \right) \, /{\text{ sample weight}}} \right] \, - \, \left[ {\left( {A_{230} /518 \times F} \right) \, /{\text{sample weight}}} \right]$$

where the A_281.5_ is absorption at wavelengths of 281.5; A_230_ is the absorption at wavelengths of 230; F is the factor for dilution in ethanol at 290 and 518 cm^−1^ wavelengths; the sample weight was 38.5 mg.

### Heat shock protein 90 expression pattern

The expression pattern of the *HSP90* gene was evaluated by a two-step RT-qPCR^25^. Following the 0, 90, 120, 150, 180, and 210 s of CAP treatment, samples were served as inoculum for the cultivation of fungus on the Sabouraud dextrose agar (SDA) medium (Merck, Germany) at 28 ºC for 9 days. Then, RNA was extracted from the samples using the TOPAZ GENE RESEARCH kit as per manufacturer instructions (Topaz gene research, IRAN). For *HSP90* gene amplification, a novel set of primers was designed with Gene Runner software (Table 2). The cDNA was then synthesized employing Parstous Easy cDNA Synthesis Kit according to the manufacturer protocol (Parstous, Iran).

**Table 2:** Primer sequences used for quantifying the expression levels of *HSP90* gene in *T. rubrum.*

Genes	Primer Sequences (5′ → 3′)	Amplicon size (bp)	Temperature	Reference
*Hsp90*	Forward: 5′-CGAGCTCTCAGACGACTCTGAC-3′ Reverse: 5′-ACCCTAGAGTTGCGATCTCATG-3′	128	62	This study
*β-actin*	Forward: 5′-TCTTCGAGACCTTCAACGCC-3′ Reverse: 5′-AAGCCACCGATCCAGAC-3′	800	58	44

The synthesized cDNA was used as a template for the RT-qPCR assay. The RT-qPCR was done with a real-time-PCR instrument (Stratagene mx3000p, USA). The reaction reagents were as follows: 6.25 µL of SYBR Green, 1 μL forward primer, 1 μL reverse primer (Table 2), 2 µL of template cDNA, and 3.25 µL of ultra-pure water to the final volume of 12.5 μL. The thermal cycler protocol included an initial denaturation at 95 ºC for 4 min, followed by 35 cycles of denaturation (94 ºC for 30 s), annealing (58 ºC and 62 ºC for 30 s), and extension (72 ºC for 30 s). The amplified products were then examined by agarose gel 2% electrophoresis to ensure the recovery of products of the expected size (128 bp). The gel image was taken by GEL DOC. The relative gene expression was calculated by the Livak method. The cycling thresholds (Ct) of the samples were normalized to β-actin Ct as calibrator and internal control.

The PCR product of the *HSP90* region was sequenced by the ABI PRISM BigDye Terminator Cycle Sequencing Ready Reaction Kit. The forward and reverse sequences of *T. rubrum* were subjected to ClustalW pairwise alignment using the MEGA7.0.21 software and edited manually to improve the alignment accuracy. The sequence of *HSP90* gene of *T. rubrum* was deposited at NCBI GenBank repository under the accession number OM994407.

### Scanning and transmission electron microscopy

Morphological changes of fungal structures were assessed in CAP-treated and untreated samples using scanning (SEM) and transmission (TEM) electron microscopy^39^. CAP-treated fungal conidia of 210 s (2 × 10^6^ cells/mL) were separately incubated in a 96-well microplate for 24 h and 96 h at 37 ºC. Fungal mat was transferred to 1.5 mL Eppendorf tubes and centrifuged to precipitate the cells. Cell pallet was kept in glutaraldehyde solution (Agar Scientific, UK) for 24 h and then washed with Phosphate buffer solution (PBS). The cells were fixed and stained by osmium tetroxide (OsO_4_) (Agar Scientific, UK), dehydrated by graded ethanol series (25, 50, 70, and 96%) and finally immersed in absolute ethanol for one hour. For SEM analysis, the final drying was performed at room temperature by hexamethyldisilazane (Merck, Germany) for 20 min to reduce the surface tension of the specimens. Specimens were air-dried for 24 h at room temperature. Sputter coating of the samples was carried out with gold (Au) to prevent surface charging of the samples. SEM images were obtained by Field-Emission SEM (FEI ESEM, Quanta 200, 3 kV, 10.0 mm). For TEM analysis, samples were post-fixed by OsO4 and embedded in epoxy resin (Agar Scientific, UK) overnight. Samples were sectioned using Ultra-microtome (MICROTOME BRAND) and stained with 2% uranyl acetate (Agar Scientific, UK) and 4% lead citrate (Agar Scientific, UK). The transmission electron microscopy images were taken by EM208S (Philips, 100 kV).

### Antifungal susceptibility testing of *T. rubrum* exposed to CAP

#### Disk diffusion method

One-hundred μL of *T. rubrum* conidia suspension (2 × 10^6^ cells/mL) was exposed to CAP in 96-well microplates for 0, 90, 120, 150, 180, and 210 s. Ten μL of CAP-exposed samples were dropped directly onto the surface of the Muller-Hinton agar plates (10 cm diameter; E-Merck, Germany), dispersed by a sterile glass rode and allowed to absorb for 5 to 10 min. Ketoconazole (KTZ; 10 μg) (Mast Diagnostics, Germany) and terbinafine (TER; 10 μg) (Mast Diagnostics, Germany) standard disks (6.0 mm Dia.) were applied separately onto the center of inoculated plates. Inhibitory zone diameters were measured at the transitional site where growth abruptly decreased after 7 days of incubation at 28–30 °C^40^.

#### Microbroth dilution method

According to M38-A2 document of the Clinical Laboratory Standard Institute (CLSI) for broth microdilution method, the minimum inhibitory concentration (MIC) of two antifungal drugs namely terbinafine (TER) and ketoconazole (KTZ) was measured. The antifungal stock solution was prepared from a commercially available antimicrobial solution. The drug concentrations and dilutions were determined based on the following formula: Volume of diluents (mL) = Weight (mg) × Potency (mg/g)/Concentration (mg/L). The test solution of the fungus was prepared adapting the same method employed in the previous section, except for the incubation time and temperature (35 °C for 96 h)^41^. MIC was considered as the least convergence of the terbinafine (TER) and ketoconazole (KTZ) that delivered a noticeable diminishing in correlation with sans drug controls.

### The MTT assay

The colorimetric MTT assay (3-(4, 5-dimethyl thiazol-2-yl)-2, 5-diphenyl tetrazolium bromide)) was used to evaluate the metabolic activity of the cells after CAP irradiation. 24 h after exposure, 20 μL of MTT solution (5 mg/mL) was added to 180 μL of CAP-exposed samples. The solutions were then incubated at 37 ºC for 4 h. The produced formazan was then solubilized by isopropanol and the optical density of the purple solution was recorded at 570 nm and 630 nm wavelengths by spectrophotometry device (EZ 301, USA)^42,43^.

## Data Availability

The datasets generated and/or analysed during the current study are available in the NCBI GenBank repository under the accession number OM994407 for *HSP90* gene of *T. rubrum*.
